# Distribution of Iron Oxide Core-Titanium Dioxide Shell Nanoparticles in VX2 Tumor Bearing Rabbits Introduced by Two Different Delivery Modalities

**DOI:** 10.3390/nano6080143

**Published:** 2016-08-03

**Authors:** Tamer Refaat, Derek West, Samar El Achy, Vamsi Parimi, Jasmine May, Lun Xin, Kathleen R. Harris, William Liu, Michael Beau Wanzer, Lydia Finney, Evan Maxey, Stefan Vogt, Reed A. Omary, Daniele Procissi, Andrew C. Larson, Tatjana Paunesku, Gayle E. Woloschak

**Affiliations:** 1 Radiation Oncology Department, Radiology Department and Pathology Core Facility, Robert H. Lurie Comprehensive Cancer Center, Feinberg School of Medicine, Northwestern University, Chicago, IL 60611, USA; tamerabdelrhman2012@u.northwestern.edu (T.R.); derek.west@northwestern.edu (D.W.); v-parini@northwestern.edu (V.P.); jasmine.may@northwestern.edu (J.M.); LunXin2015@u.northwestern.edu (L.X.); k-r-harris@northwestern.edu (K.R.H.); will.c.liu@gmail.com (W.L.); m-wanzer@northwestern.edu (M.B.W.); d-procissi@northwestern.edu (D.P.); a-larson@northwestern.edu (A.C.L.); tpaunesku@northwestern.edu (T.P.); 2 Clinical Oncology Department and Pathology Department, Faculty of Medicine, Alexandria University, Alexandria 21131, Egypt; samarelachy@yahoo.com; 3 X-ray Sciences Division, Advanced Photon Source, Argonne National Laboratory, Argonne, IL 60439, USA; lfinney@aps.anl.gov (L.F.); emaxey@aps.anl.gov (E.M.); svogt@aps.anl.gov (S.V.); 4 Department of Radiology and Radiological Sciences, Vanderbilt University School of Medicine, Nashville, TN 37232, USA; reed.omary@vanderbilt.edu

**Keywords:** transarterial intra-catheter delivery, core–shell nanoparticle, rabbit VX2 liver cancer model

## Abstract

This work compares intravenous (IV) versus fluoroscopy-guided transarterial intra-catheter (IC) delivery of iron oxide core-titanium dioxide shell nanoparticles (NPs) in vivo in VX2 model of liver cancer in rabbits. NPs coated with glucose and decorated with a peptide sequence from cortactin were administered to animals with developed VX2 liver cancer. Two hours after NPs delivery tumors, normal liver, kidney, lung and spleen tissues were harvested and used for a series on histological and elemental analysis tests. Quantification of NPs in tissues was done both by bulk inductively coupled plasma mass spectrometry (ICP-MS) analysis and by hard X-ray fluorescence microscopy. Both IV and IC NPs injection are feasible modalities for delivering NPs to VX2 liver tumors with comparable tumor accumulation. It is possible that this is an outcome of the fact that VX2 tumors are highly vascularized and hemorrhagic, and therefore enhanced permeability and retention (EPR) plays the most significant role in accumulation of nanoparticles in tumor tissue. It is, however, interesting to note that IV delivery led to increased sequestration of NPs by spleen and normal liver tissue, while IC delivery lead to more NP positive Kupffer cells. This difference is most likely a direct outcome of blood flow dynamics. Armed with this knowledge about nanoparticle delivery, we plan to test them as radiosensitizers in the future.

## 1. Introduction

Hepatocellular carcinoma (HCC) is emerging as a public health problem worldwide as the third most common cause of cancer-related deaths [1] and secondary metastatic liver tumors are even more frequent than primary hepatocellular cancer disease. Transarterial embolization (TAE) and transarterial chemoembolization (TACE) have been established as effective treatment modalities for unresectable liver tumors; these approaches are currently also investigated as an alternative to surgery in potentially resectable tumors [2]. The possibility of liver tumor treatments by nanomaterials is currently under investigation, as well as the idea that nanoparticles’ accumulation in liver may be useful for treatments of other liver diseases. This idea constitutes one of the US National Cancer Institute (NCI)’s “provocative questions” for use of nanomaterials in cancer research [3].

In this work, we focused on the VX2 induced carcinoma implanted in the rabbit liver as a well-established model for liver tumors. This model system has been in use for over two decades, especially in radiology because these tumors can be imaged by the same approaches and with the same equipment used for humans [4]. One type of study that can be done with this animal liver cancer model is to compare intravenous (IV) versus transarterial intra-catheter (IC) delivery of contrast agents; our group as well as others have focused on these comparative studies [5,6,7,8,9,10,11]. In this work we have used for the first time the same two delivery approaches to introduce a novel type of core–shell nanoparticle (Supplemental Figure S1) into VX2-bearing rabbits. This nanoparticle formulation was investigated in vitro as a carrier for delivery of doxorubicin to doxorubicin-resistant cells [12] and as a source of cytotoxic reactive oxygen species following nanoparticle excitation [13]. Specifically, core shell nanoparticles were found to produce more reactive oxygen species after excitation than pure TiO_2_ [13]. Either mode of application can be used therapeutically after an optimal distribution pattern has been confirmed, thus, this work forms the basis for therapy testing efforts in the future. While work of others focused on other modes of nanoparticle delivery, e.g., use of magnetic field to fortify nanoparticle deposition in tumors [9], we are trying to investigate success of nanoparticle distribution when they are decorated by cortactin peptide and injected into the bloodstream via IV or IC. In this work, we investigated NP accumulation in VX2 tumors as well as in healthy parts of the liver, lung, kidney and spleen. Moreover, we examined these tissue samples closely, giving special attention to the accumulation of NPs in Kupffer cells as representatives of liver reticuloendothelial system (RES). We have also included in this study an investigation of cell proliferation and apoptosis in VX2 tumors and other tissues using immunohistochemistry.

## 2. Results

This study included a total of eight rabbits; three rabbits received NPs intravenously (IV) by injections into ear vein (10 mL NP volume), three rabbits received transarterial intra-catheter (IC) injection (3 mL NP volume) and two control rabbits with liver VX2 tumors untreated with NPs. Difference between injection volumes was motivated by the attempt to deliver maximal nanoparticle volume for a given administration route. Thus, for the intravenous injection (IV), we used 10 mL nanoparticle volume, a value close to maximal tolerated for a rabbit ear vein injections. Likewise, for the transarterial intra-catheter (IC) nanoparticle delivery, direct injection into liver was done with a lower volume of liquid—3 mL, again the most that can be tolerated as IC by the rabbits (Supplemental Figure S2).

**Figure 1:**
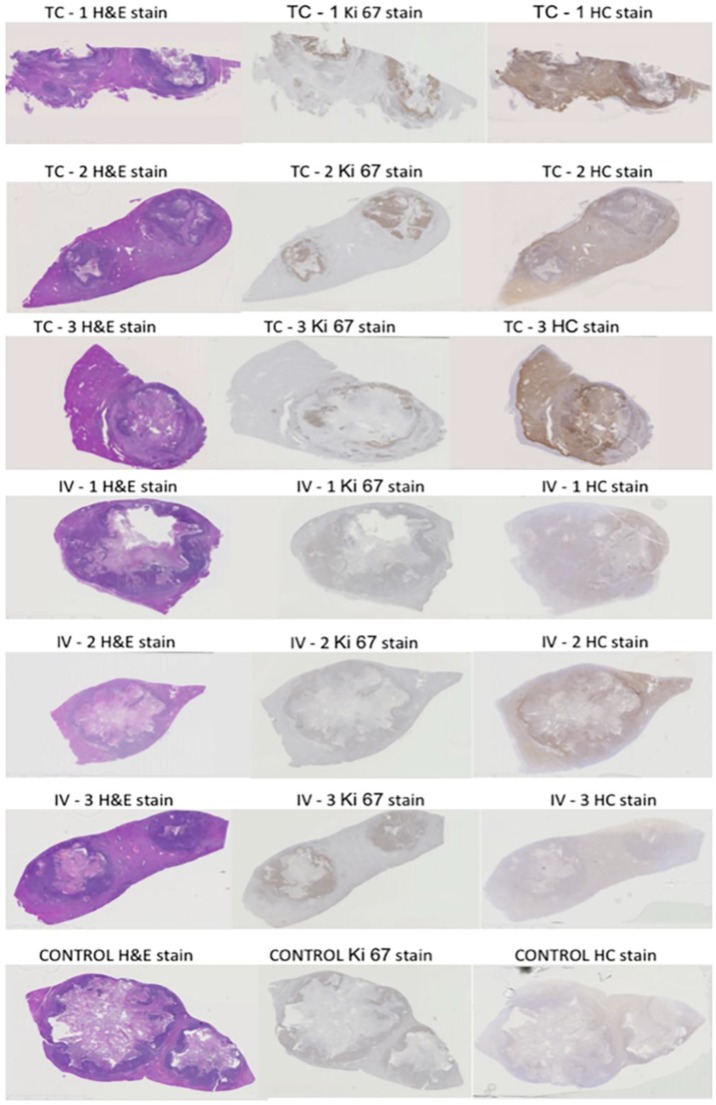
Intravenous (IV) and transarterial intra-catheter (IC) injected rabbits’ VX2 tumors and surrounding liver tissue stained by H&E, histochemical (HC) stain (detecting the nanoparticles) and Ki67 Antibodies.

Figure 1 shows overview micrographs of the tissues stained by hematoxylin and eosin (H&E), dopamine stain for nanoparticle distribution and immunohistochemistry (IHC) for Ki67. The viable tumor area indicated by the dense distribution of cell nuclei, overlaps with the most intense Ki67 staining and the darkest NPs distribution stain (Figure 1), suggesting that nanoparticle accumulation is greatest in the viable region of the tumor.

**Figure 2:**
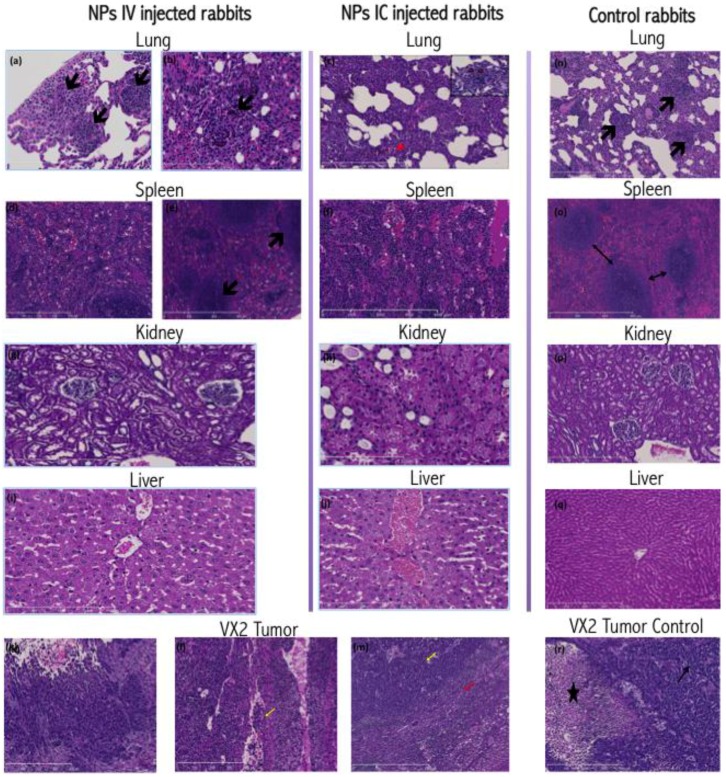
Details from Supplemental Figure S3. (**a**) IV injected rabbit’s lung showing metastatic nodular tumor deposits disposed in a peripheral location composed of poorly differentiated squamoid cells expanding the alveolar septae (100× H&E); (**b**) A photomicrograph of the IV injected rabbit lung showing alveolar septae expanded and infiltrated by inflammatory infiltrate, predominantly eosinophils (200× H&E); (**c**) IC rabbit lung showing marked thickening and destruction of alveolar spetae by metastatic deposits and inflammatory infiltrate composed mainly of granulocytes (arrowheads in inset, 400×). Blood vessels are engorged with granulocytes (arrow head) (100×); (**d**,**e**) IV injected rabbit’s splenic red pulp displaying dilated and congested sinusoids. Arrows point to follicles (100× H&E); (**f**) IC injected rabbit spleen showing congested and expanded red pulp with influx of macrophages and proliferated Littoral cells; (**g**) IV injected rabbit renal cortex showing mild to moderate cloudy changes in the tubules (100× H&E); (**h**) IC injected rabbit kidney showing cortical tubules exhibiting severe cloudy swelling and vesicular changes (100× H&E); (**i**) An IV injected rabbit liver micrograph displaying mild lobular disarray with hepatocyte ballooning (200× H&E); (**j**) IC injected rabbit’s liver showing dilated congested central veins and sinusoids. Hepatocytes show ballooning and microvesicular steatosis (200× H&E); (**k**) Photomicrograph showing part of VX2 tumor mass with a central necrotic cavity (100× H&E); (**l**) A photomicrograph of VX2 tumor showing infiltrating malignant cells on the left side, tumor infiltrating lymphocytes on the right, and a dilated sinusoid with tumor thrombus in the center (arrow) (100× H&E); (**m**) Photomicrograph of VX2 tumor showing nests of squamous cell carcinoma (yellow arrow), surrounded by a peripheral zone of tumor infiltrating lymphocytes (red arrow), compressed hepatocytes and fibroblasts; (**n**) Control rabbit lung micrograph showing multiple microscopic metastatic tumor deposits (arrows), congested microvasculature and patent alveoli (100× H&E); (**o**) Control Rabbit spleen micrograph showing white pulp follicles with narrow intervening red pulp sinusoids, minimal congestion ascribed to manipulation during necropsy; (**p**) Control rabbit kidney micrograph showing patent cortical tubules and unremarkable glomerular tufts with absence of congested vessels (100× H&E); (**q**) Photomicrograph of control rabbit liver (100× H&E); (**r**) Control Rabbit micrograph of VX2 liver tumor showing malignant squamoid cells (arrow) surrounding a central necrotic cavity (star) (100× H&E).

In all animals included in this work, histopathologic examination of VX2 tumors revealed irregular infiltrative tumor masses with large central necrotic cavities composed of poorly differentiated, highly pleomorphic squamoid cells disposed in the form of nests, trabeculae and solid sheets (Figure 2).

Other tissues harvested from tumor-bearing rabbits displayed few changes. The healthy liver tissue from all animals showed mild to moderate centrilobular hepatocyte ballooning and microvesicular steatosis along with focal drop out necrosis. Kupffer cell hyperplasia in the livers was noticed, possibly associated with inflammation, which has been noted in VX2 carrying rabbits [7]. Livers from IC injected animals exhibited more congested central veins and centrilobular sinusoidal dilation.

Rabbit lungs revealed a picture of interstitial pnuemonitis with marked thickening and destruction of the alveolar septae, bearing multiple metastatic tumor deposits and inflammatory infiltrate (neutrophils, esinophils and macrophages), and congested vasculature. The spleens of rabbits with VX2 tumor burden revealed mildly congested and dilated red pulp sinusoids.

**Figure 3:**
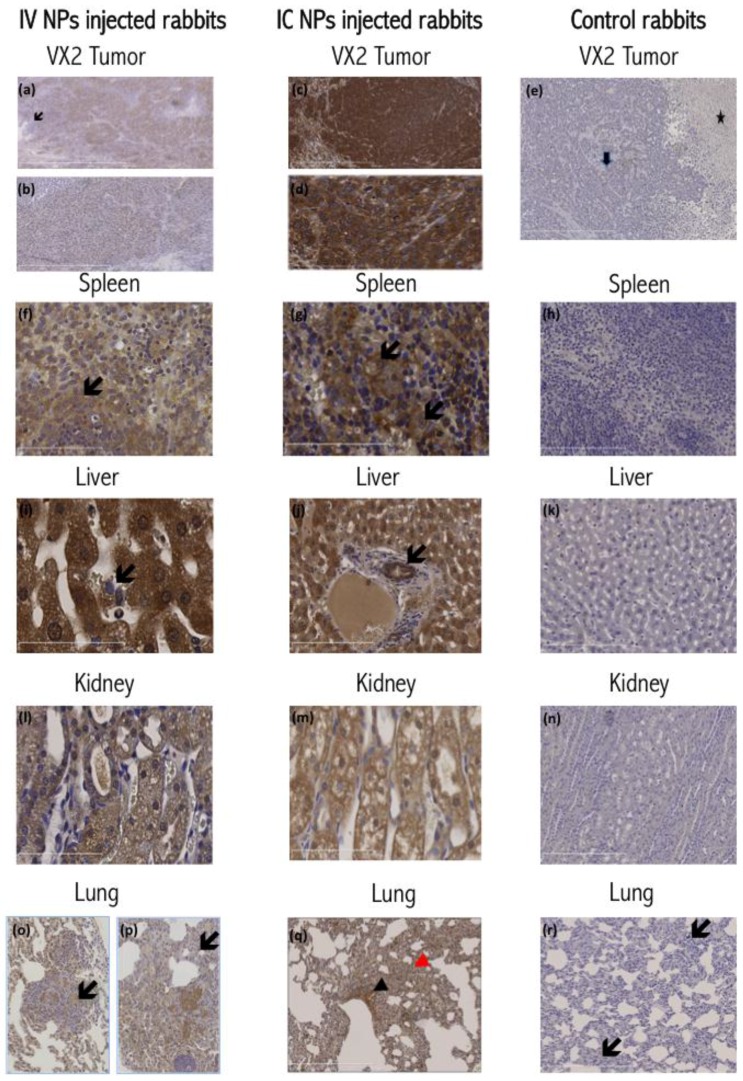
Details from Supplemental Figure S3. Histochemical Staining for NPs: (**a**) IV injected rabbit’s VX2 tumors showing weak uneven staining of tumor masses. Arrow shows weak staining of surrounding hepatocytes (100×); (**b**) Photomicrograph showing nuclear staining of IV injected rabbit’s VX2 tumor cells with weak cytoplasmic stain. Surrounding tissues are not stained (200×); (**c**) Intense brown nuclear and cytoplasmic staining of the IC injected rabbit’s tumor masses (100×); (**d**) Micrograph of IC injected rabbit’s VX2 tumor showing intense positive nuclear and cytoplasmic staining of cells (400×); (**e**) Photomicrograph of control rabbit VX2 showing faint non-specific staining of malignant tumor cells (arrow) and necrotic tissues (star); (**f**) Micrograph of IV injected rabbit’s spleen showing positively stained red pulp macrophages and marginal zone macrophages (arrows) (200×); (**g**) Micrograph of IC injected rabbit’s spleen showing positively stained red pulp macrophages (400×); (**h**) Micrograph of control rabbit’s spleen red pulp showing absence of positive staining (200×); (**i**) Positively stained Kupffer cell in a sinusoid of an IV injected rabbit (400×); (**j**) IC injected rabbit’s micrographs showing intense staining of periportal hepatocytes and bile duct epithelium (arrow) (200×); (**k**) Micrograph of control liver showing no positive staining (200×); (**l**) IV injected rabbit renal medullary tubules showing intense positive cytoplasmic stain (200×); (**m**) IC injected rabbit’s micrograph of rabbit medullary tubules of kidney showing intense positive cytoplasmic stain (200×); (**n**) Control rabbit’s kidney micrograph showing no positive staining (100×); (**o**) IV injected rabbit’s lung showing a nodular metastatic deposit with positively stained tumor cells (200×); (**p**) IV injected rabbit’s lung displaying positively stained tumor metastatic deposits, in addition to alveolar macrophages. Arrow is pointing at a positively stained alveolar macrophage (200×); (**q**) IC rabbit lung showing positive brown staining of metastatic deposits within the alveolar septae (red arrowhead) and the bronchiolar epithelial lining cells (black arrowhead) (200×); (**r**) Micrograph of control rabbit’s lung showing absence of positive staining in metastatic deposits (arrows) and alveolar macrophages (100×).

Histochemical staining for nanoparticle distribution (Figure 3) in the IC injected VX2 tumors revealed an extensive, homogenous, dense brown staining of the tumor cells, evident both in cytoplasmic and nuclear regions of cells. This staining showed reduced density towards the center of the tumor and farthest from the blood supply and the viable tumor cell region. In IV injected rabbits, NP staining pattern demonstrated irregularities, with an uneven staining density of the tumor cells, and some areas very positive, while in others the staining was extremely weak. In both groups, the peritumoral hepatocytes, fibroblasts and lymphoplasmacytic infiltrates show no or minimal staining, suggesting that nanoparticle accumulation in these cells is minimal.

The rabbit livers derived from IV-injected animals demonstrated brown cytoplasmic positivity in centrilobular hepatocytes as well as the Kupffer cells. However, IC injected rabbits display a different pattern of nanoparticle distribution. In these animals, the more intensely stained Kupffer cells and hepatocytes reside in the periportal zones of the lobule, and positive staining of the bile duct epithelia can be observed as well.

The spleens of all NP injected rabbits (Figure 3) showed positive cytoplasmic staining in the red pulp and sinusoidal macrophages. However, the IV injected rabbits showed positively stained marginal zone macrophages of the white pulp. The kidneys of all NP injected rabbits revealed positive cytoplasmic staining of the renal tubules, more intense in the medullary tubules. Metastatic tumor deposits residing within the lungs of both groups invariably included positively stained tumor cells. Additionally, alveolar macrophages and bronchial epithelial cells also displayed cytoplasmic positivity suggesting presence of nanoparticles (Figure 3). Histochemical staining of control rabbit tissues showed no or minimal positive brown stain.

**Figure 4:**
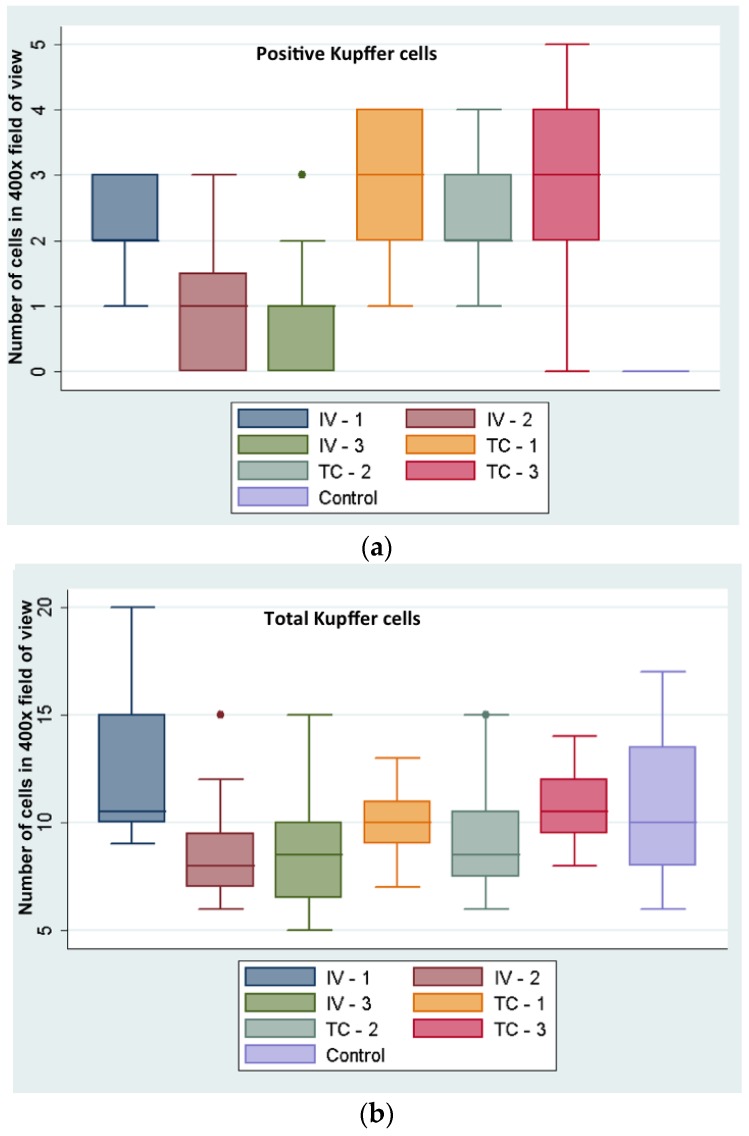
(**a**) NP positive Kupffer cells and (**b**) total number of Kupffer cells in livers of intravenous (IV) and intra-catheter (IC) injected and control rabbits. *Y*-axis indicates total number of Kupffer cells per field of view, averaged from 20 such areas, images acquired at 400× (for example, see one such area of view in Figure 3i).

Hepatic Kupffer cell index (NPs positive Kupffer cells in 20 high power fields (HPFs) (at 400× magnification) divided by the total number of Kupffer cells in 20 HPFs was higher in IC-injected (28%) versus the IV-injected rabbits (13%). While this difference appears as statistically significant (*p* = 0.005) by Student’s *t*-test, the numbers of NP containing Kupffer cells are too low for a meaningful conclusion (Figure 4).

**Figure 5:**
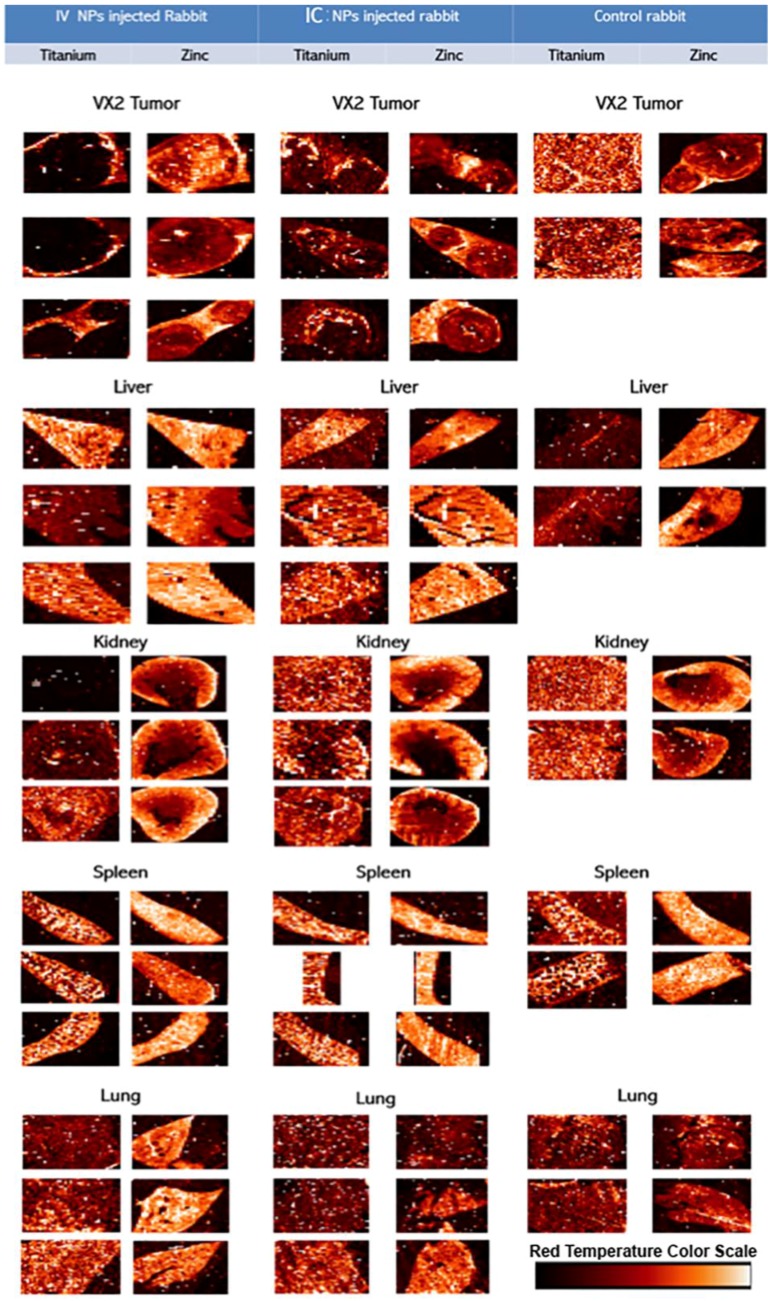
Overview of XFM images for NP distribution after intravenous (IV) and transarterial intra-catheter (IC) injections into control rabbits VX2 tumor and liver, kidney, lung, and spleen tissues. The mapping for the Titanium (**left**) and Zinc (**right**) concentrations and content informs about tissue shape, and potentially even its health status. It should be noted that these are false color images showing distribution of elemental concentrations from the lowest (**black to brown**) to highest (**yellow to white**) within the elemental concentrations distribution of each sample (see color scale bar in lower right depicting spectra of colors matching lowest concentration (in a given sample)—black) to highest (**white**) across a “red temperature” scale. Thus, samples with very low elemental concentrations for a given element (e.g., Ti in control samples) show a “salt and pepper” pixel distribution indicating that background signal levels predominate in the sample. It should be noted that paraffin embedding disrupts distribution of free ions such as potassium, while sulfur and zinc distributions still well represent a tissue outline because Zn finger proteins are accumulated in each cells’ nucleus [14,15,16,17,18,19]. Distribution of nanoparticles, much larger and insoluble *in situ*, is considered to be accurately depicted by XFM [14,15,16,17,18,19].

**Figure 6:**
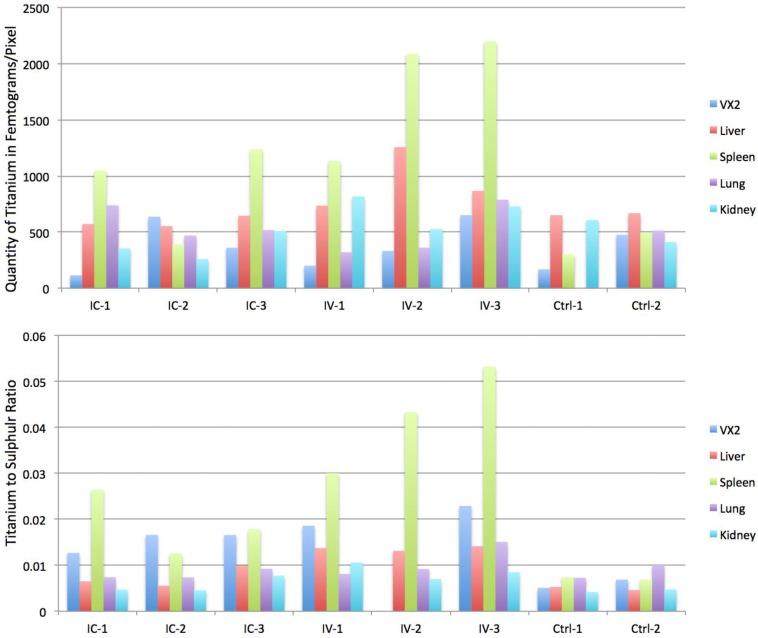
Graphic representation of data in Table 1. (**Top**) Ti concentration (in femtograms per pixel) in different tissues from nanoparticle treated animals and controls; data for each animal are shown. (**Bottom**) Mean ratio of Ti (femtograms) vs. S (femtograms) per pixel; please note that data structure allows comparison on a per pixel basis. Thus, information provided by the bottom graph allows us to monitor the situation most accurately, e.g., if tissue integrity is uneven, resultant uneven concentration of nanoparticles in tissue will not be misinterpreted. Likewise, false background (“salt and pepper” signal pattern for Ti) is effectively removed when false signals are divided by real signal for S.

Hard X-ray fluorescence microscopy (XFM) was used for mapping of elemental content of these tissues (Figure 5); followed by a “region of interest” ROI analysis. ROIs were drawn around different regions of the image. In healthy tissue sample images ROIs were drawn to match the tissue vs. background; in tumor samples ROIs were drawn to segregate the viable area of VX2 tumor from the tumor necrotic core and from the region corresponding to healthy liver tissue. Subsequent quantitative analysis of regions of interest was done to calculate the ratio of Ti content against zinc, sulfur and phosphorus content. When ratios were significantly different for all of thre pairs of elements (and not significant for the control Ti/Fe ratio) that organ was considered to be differentially affected by the route of nanoparticle delivery. All concentrations were obtained in femtograms (10^−15^ g) per unit area. Statistically significant ratiometric increase of Ti vs. native tissue elements was noted in IV injected rabbits compared to the IC injected animals when healthy tissue are considered. In short, ratios of Ti/Zn, Ti/S and Ti/P were found to be increased in spleen (*p* < 0.04) and healthy portions of the liver (*p* < 0.03) while no statistically significant elemental ratio differences were found in the kidney, lungs or VX2 tumor itself (note, however, that 10 mL of NP was injected IV while only 3 mL of NP was administered IC) (Table 1, Figure 6).

The accumulation of titanium in different organs and VX2 tumors was also evaluated by ICP-MS. Due to the large bulk of the rabbit tissue we did not digest complete organs, but limited ourselves to digests of small volumes of tissue and calculating the elemental content against tissue weight. Ti quantity per milligram of tissue was highest in the spleens, with a statistically significant difference (*p* = 0.014) between the concentrations of Ti in spleens of IV (mean = 13.959 ppb/mg of tissue, SD = 4.067) vs. IC injected rabbits (mean = 3.647 ppb/mg of tissue, SD = 1.198). There was also a statistically significant difference between the concentrations of Ti in VX2 tumor tissues in IV injected rabbits (mean = 0.623, SD = 0.099) vs. IC injected rabbits (mean = 2.755, SD = 0.782). For this measurement only the viable region of the tumor was used. The normal liver parenchyma also showed a statistically significant difference in Ti accumulation (*p* = 0.00107). The mean Ti concentration in the IV NPs injected group was 4.89 ppb/mg (SD = 0.526) vs. 2.03 ppb/mg (SD = 0.255) in the IC NPs injected group. There was no statistically significant difference (*p* > 0.09) in the ICP-MS data regarding the concentrations of Ti in kidneys or lungs (*p* = 0.089).

Immunohistochemistry for Ki67 was done for VX2 tumor tissue only. No statistically significant difference between the two groups of NP injected rabbits was found (*p* > 0.2), suggesting that NP accumulation differences resulted from different routes of injection and not from structural differences between the tumors.

Furthermore, no statistically significant difference was found between the two groups regarding the Terminal Deoxynucleotidyl Transferase (TdT)-Mediated dUTP Nick-End Labeling (TUNEL) apoptosis index (*p* = 0.2).

## 3. Discussion

In this work, we have used the VX2 rabbit liver tumor as a model system for hepatocellular carcinoma. This model system has many convenient features suitable for translational research; most notably, diagnostic imaging modalities (ultrasound, CT, MRI), and equipment suitable for work with humans can also be used for rabbits, while that is never the case with more traditional rodent animal models. The VX2 tumors receive their blood supply almost exclusively from the hepatic artery, which simulates human HCC. Furthermore, the relatively wide diameter of rabbit hepatic arteries facilitates hepatic artery catheterization [7]. Although other larger animals could be used in a similar way—for example some reports recommend the eastern woodchuck HCC model as the best model for human HCC; we used the rabbit VX2 tumor model in this work for the following reasons: (i) immunohistochemistry tools for work with rabbit tissues are better developed than those for the woodchuck system; and (ii) the primary focus of this study is on NPs fate with different routes of NP delivery, and we have many years of experience with the IV and IC delivery in rabbit VX2 model [5,6,7,8,10,11,20].

This study aimed to investigate the VX2 liver tumor model as a testing ground for cytotoxic ultrasmall nanoparticles with TiO_2_ shell developed by our laboratory [12,13,14,15,16,17,18,19,21,22,23]. It should be noted that others have used VX2 models to test other types of nanoparticle formulations, e.g., magnetic particles that could be directed by magnetic field to accumulate in hind limb tumors and deliver therapeutic cargo [9]. In this work we used approaches well developed by interventional radiology: IV and IC delivery routes; we compared the success of NP delivery to VX2 tumor cells. The study showed comparable success with regard to NP accumulation in the viable region of the tumor using both techniques. Even though IC delivery was slightly more efficient (accumulation of Ti was within the same order of magnitude for both delivery approaches using ICP-MS or XFM), this difference is not high enough to justify abandoning the IV approach because IV delivery route is less invasive, requires fewer resources and is less time consuming. However, NPs accumulation in non-target tissues could be a reason to use IC approach rather than the IV, and a more extensive study conducted with the VX2 bearing animals will have to be done in order to evaluate the potential toxicity of NPs [19]. Our experiments documented differences in NPs accumulation at 2 h after NPs delivery. Both ICP-MS and XFM analyses show that the accumulation of Ti was highest in the spleen especially in IV injected rabbits.

This work shows that NPs accumulation evaluation can be done either by ICP-MS analyses or by high throughput XFM imaging. In both cases, however, non-homogeneous NPs distribution in different tumor and tissue regions complicates NPs accumulation interpretation. For that reason, XFM may be better suited for investigation of inhomogeneous NPs distribution. For example, XFM can be used to map the elemental content of tissues at very high resolution, in single cells [24]; and the detection of Ti containing nanoparticles is made easier due to the absence of Ti background in cells and tissues [14,15,16,17,18]. The value of ICP-MS is the greatest when the entire tissue can be digested and analyzed, while XFM can be used to gain an overview of elemental distribution for many elements at the same time and allow inspection of non-homogeneous elemental distribution caused by such issues as alterations in tissue vasculature and variable blood supply in the different tumor areas.

However, in our study, XFM analysis demonstrated no statistically significant difference regarding Ti total content in the VX2 tumor in either group. It should be noted that only 3 mL was injected IC versus 10 mL IV, due to differences in injection volume tolerance dependent on location of delivery. Therefore, similar nanoparticle accumulation suggests that the VX2 tumor accumulation of NP at 2-h post injection was more efficient in case of IC delivery. In addition, the higher concentration of Ti in the spleen, liver and kidneys of IV injected animals suggests that there are likely to be more “off-target” effects with IV injection of nanoparticles. Therefore, choice between IV and IC delivery will have to be weighed between benefits of lowering the complications inherent in IC delivery vs. benefits of off-target accumulation of nanoparticles in spleen and liver.

Finally, one also needs to consider that the nanoparticles used in this study were surface modified by addition of peptide sequence from protein cortactin, responsible for binding to potassium ion channel protein Kv1.2 [25,26]. This selection of targeting agent was inspired by the knowledge that K^+^ channels play a significant role in cancer development and progression [27]. By adding a targeting agent to NPs we hoped to improve delivery of NPs when administered IV. While accumulation of NPs with both delivery methods was comparable, at least based on XFM evaluation, deposition of NPs to non-target tissues was still higher in IV treated rabbits. This knowledge about nanoparticle distribution will allow us to plan subsequent experiments where nanoparticles will be used as radiosensitizers or vehicles for delivery of chemotherapeutic drug cargo.

## 4. Materials and Methods

### 4.1. Animal Model and Tumor Induction

This study included ten New Zealand white rabbits weighing between 12 and 20 lbs: two donor rabbits (with hind limb tumors), two control animals (with VX2 tumors implanted in liver but not treated with nanomaterials), and six test animals with liver implanted VX2 tumors. Of the latter, three received NPs by IV injection and three by IC injection. The institutional animal care and use committee of Northwestern University approved all work.

VX2 cells, originally procured from National Cancer Institute (NCI, Frederick, MD, USA) [14] were injected in the hind limb of a donor rabbit and allowed to grow for three to four weeks. The rabbit was scanned regularly using ultrasound to evaluate tumor growth; when the growth reached 1–2 cm the donor animal was sacrificed and the tumors (as well as “control tissues”) were harvested. The hind limb tumor was dissected and the central necrotic region removed. Viable tumor tissue was then minced and suspended in sterile Hank’s solution.

Eight rabbits had their livers surgically implanted with minced VX2 tumor tissue. Anesthesia in animals was induced by intramuscular injection of ketamine at 44 mg/kg and xylazine 3–5 mg/kg, and the rabbit was maintained under inhalational isoflurane at 2%–3% during the surgical procedure. A laparotomy incision, usually 5–7 cm in length was made, starting from the tip of the xiphoid process downwards. The liver was exposed, and 0.5 mL each of viable tumor tissue was injected into the liver in two locations in the same liver lobe. A sharp blade was used to induce small incisions in the liver capsule prior to injection; absorbable plaster was used to cover the incisions. The abdomen was then closed; the muscles layer was sutured in two levels as well as the skin using subcuticular sutures. After the surgery rabbits were monitored closely during the first 48 h postoperatively. Two weeks post-surgery, the tumor growth was monitored using 7T magnetic resonance imaging. This work followed closely procedures long established [5,7,8,10,11,20] by our laboratory.

### 4.2. Nanoparticle Preparation

Core–shell Fe_3_O_4_@TiO_2_ nanoparticles were synthesized as described [12,13,19]; In short, this procedure begins with production of ultrasmall (2–3 nm) Fe_3_O_4_ nanoparticles that are used instead of water into which TiCl_4_ is added dropwise, following approaches we used for synthesis of pure TiO_2_ nanoparticles [14,15,16,17,21,23]. Nanoparticles were coated with glucose in a process of 48 h long dialysis in appropriate buffer (10 mM sodium phosphate, 0.9% sodium chloride and 4% glucose). Concentration of Ti and Fe in nanoparticle solution was determined by ICP-MS on X Series II Inductively Coupled Plasma-Mass Spectrometer (Thermo Scientific, West Palm Beach, FL, USA). A series of standards was used, from 0 ppb to 50 ppb Ti or Fe, with 3 ppb of indium spike in the samples and standards serving as an internal control. Calculation of NP and NP surface sites molarity was based on elemental concentration determined by ICP-MS and an estimate of nanoparticle size based on cryo TEM images (Supplementary Figure S1). Idealized uniform and spherical shape was used as basis for these calculations, similar to work done before [12,13,14,15,16,17,18,19,21,22,23]. Nanoparticle concentration was determined as 0.87 μM (that is, approximately 5.23 × 10^17^ nanoparticles per liter) with size distribution centered around 12 nm and zeta potential between −30 and −50 mV depending on surface modifications. Nanoparticles aggregated into structures of up to 50nm (see Supplementary Figure S1). Cumulative concentration of nanoparticle surface binding sites (for 3,4-Dihydroxyphenylacetic acid (DOPAC)) was calculated at approximately 0.5 mM using the approach described before [12]. About 30% of nanoparticle surface decorated with peptide (N terminus) GITAVALYDYQAAAK-DOPAC (C terminus), a segment of human cortactin (OMIM entry 164765) [26], isoform X1 (NCBI XP_006718510.1). Peptide synthesis with addition of DOPAC enables ready binding to nanoparticle surface. In general, molecules with a catechol group bind to the TiO_2_ nanoparticle surface avidly, and among them dopamine and DOPAC are among the most stable [12,13,14,15,16,17,18,19,21,22,23]. Peptide synthesis was done by the core facility of Northwestern University.

### 4.3. Nanoparticle Delivery

After confirmation of tumor growth by MRI, three rabbits were given IV injection of 10 mL of NPs, while another three rabbits received intra-catheter injection of only 3 mL of the same NP preparation. The intra-catheter injection was performed as X-ray digital subtraction angiography (DSA) using a Siemens C-arm Power Mobil unit (Siemens Medical Solutions, Erlangen, Germany). The three rabbits were sedated using a mixture of IM ketamine (80 mg/kg) and xylazine (5 mg/kg) and were maintained under inhalational isoflurane (2%–3.5%), with additional IM ketamine as needed. To perform the IC injection, a cut-down was performed to access the rabbit’s femoral artery. The artery was catheterized with a 3-F vascular sheath (Cook, Bloomington, IN, USA), and then a 2-F catheter (Cook JB-1) was advanced over a 0.014-inch diameter guide wire until it reached the celiac artery. A full-strength Omnipaque 350 (Amersham Health, Princeton, NJ, USA) contrast agent was injected to localize the celiac, common hepatic, or left hepatic arteries. Once we confirmed the accurate localization of the catheter in the common hepatic artery, we sutured the rabbit’s groin for placement fixation. Three milliliters of NPs were then injected trans-catheter into the tumor site. We use X-ray DSA imaging routinely to help us guide transarterial intra-catheter delivery to liver tumors [6,7,8,10,11,20]. Rabbits were then imaged by MRI (Bruker 7T ClinScan MRI System, Camarillo, CA, USA) while monitoring the respiration and heart rates right after injection of the NPs and then terminated and tissues (liver, lung, kidney, spleen and VX2 tumors) were harvested (Figure 1). While iron oxide cores of core–shell nanoparticles are superparamagnetic (data not for publication), their concentration in vivo was too low to allow these nanoparticles to serve as a contrast agent for MRI.

### 4.4. Tissue Sample Preparation

Two hours after injection of NPs, animals were sacrificed and their organs extracted. The harvested tissues: VX2 tumor, normal liver, spleen, lung and kidney were cut into portions. Larger tissue sections were fixed in 10% buffered formalin and paraffin embedded. Separate tubes with small tissue pieces were frozen and stored in −80 °C for future processing.

NP distribution in tissue sections (4 micron thick) was evaluated by visible light microscopy using a histochemical technique (see below) that specifically stains the nanoparticles. In addition, series of thicker tissue sections (7 micron thick) was placed on Ultralene membrane (SPEX CertiPrep, Metuchen, NJ, USA) and mapped for elemental distribution by high throughput hard X-ray Fluorescence Microscopy (XFM) at the 8BM-B beamline at the Advanced Photon Source at Argonne National Laboratory. The quantity of Ti, Fe and other elements in specific regions of interest was determined in femtograms using calibrated XFM 2D map data.

Small frozen tissue pieces were weighed and digested for processing by ICP-MS. The quantity of Ti and Fe per milligram of tissue was recorded as shown later in results section.

Tissues embedded in paraffin were also sectioned into 4 microns sections and stained using hematoxylin & eosin for standard histopathological examination to detect signs of inflammation, which is a typical side effect of VX2 tumor growth in liver, as well as any other signs of tissue injury. Additional tissue sections were processed for immunohistochemistry.

### 4.5. Histochemical Detection of Nanoparticle Distribution

Four microns thick tissue sections were cut from the paraffin blocks, deparaffinized and rehydrated in descending grades of ethyl alcohol. These samples were then incubated overnight in an oxygen free atmosphere in a 0.2 μM solution of biotin-conjugated to dopamine. The completeness of conjugation between dopamine and biotin was evaluated by mass spectrometry. TiO_2_ nanoparticle surface molecules have great affinity for binding catechols [12,13,14,15,16,17,18,19,21,22,23] and dopamine can readily displace glucose coat on nanoparticle surfaces, labeling the nanoparticles in situ with biotin. The slides were then washed in Dulbecco’s Phosphate-Buffered Saline (DPBS) (Mediatech, Inc., Manassas, VA, USA) and a hydrogen peroxide (H_2_O_2_) 3% block was applied for 30 min (VWR International, West Chester, PA, USA). The slides were washed again and streptavidin peroxidase SS Label (Cat. No. HK330-9K, BioGenex, Fremont, CA, USA) was applied for 30 min followed by another DPBS wash. DAB-Chromogen (Cat. No. HK130-5K, BioGenex, Fremont, CA, USA) was prepared according to manufacturer instructions and applied for 5 min. The slides were finally washed and counterstained with hematoxylin.

All stained slides were imaged using a Hamamatsu K.K. Nanozoomer 2.0 HT and examined by a blinded experienced pathologist.

Kupffer cell numbers were evaluated semi-quantitatively by manual counting of 20 random high power image fields, screening the healthy portion of liver tissues of all rabbits; nanoparticle positive cells and total numbers of Kupffer cells were counted.

### 4.6. X-ray Fluorescence Microscopy (XFM)

XFM provides spatial information regarding the elemental content of cells and tissues, and we have used this technique to monitor both native and nanoparticle treated samples [12,14,15,16,17,18,19,24]. Seven-microliter-thick paraffin tissue sections were placed on Ultralene membrane (SPEX Sample Prep, LLC, 15 Liberty St., Metuchen, NJ, USA).

Elemental mapping was done at the Advanced Photon Source Synchrotron at the Sector 8 bending magnet beamline (8BM-B, Argonne National Laboratory, Argonne, IL, USA). Samples were raster scanned with a beam width of several hundred microns in order to obtain a high throughput overview of the distribution of elements in these tissues. K alpha fluorescence was measured for all elements between potassium and zinc in the periodic table. Concentrations of phosphorus, sulfur, titanium, iron, and zinc in these samples were calculated after calibration with a National Institute of Standards and Technology standard. Elemental quantities (in femtograms) in 2D regions of interest were determined. Considering that phosphorus, sulfur and zinc are biologically relevant elements that can be used to represent quantity of biological material, ratio of titanium to sulfur etc. was used to evaluate nanoparticle accumulation differences between different samples.

### 4.7. Inductively Coupled Plasma Mass Spectrometry (ICP-MS)

ICP-MS can be used to detect individual elements, metals as well as several non-metals, in digested bulk samples at concentrations as low as one part in 10^12^ (part per trillion). We used the ICP-MS to detect the concentration of titanium and iron in selected small pieces of tissue from healthy liver, kidney, lung, spleen and VX2 tumor.

For ICP-MS sample preparation, the frozen tissues were partially thawed in ice for 20 min, additionally cut and weighed on an analytical balance. Tissue pieces (between 10 and 30 mg) were placed in metal free 50 mL tubes labeled with organ name and rabbit number. Five hundred microliters of autoclaved filter water was added to each tube followed by 128.6 μL of 70% nitric acid. The tubes were then heated in a water bath at 70 °C overnight. The tubes were cooled to room temperature and then 1 mL of filtered water was added to each tube and the solutions centrifuged for 1 min at 1500 rpm to collect the evaporation on the sides of the tube and to investigate if any pellet was present; presence of a pellet was considered as an indicator of failed digest and the tissue digestion was done with a new tissue piece. Well digested (pellet free) samples were then filtered using a 0.2 μm syringe filter and spiked with 15 uL of 0.6 μg/mL indium solution. Finally, the samples were diluted with water in a total V of 3 mL per sample. Serial dilutions of elemental (Ti or Fe) standards were prepared covering a range of concentrations between 0 and 50 parts per billion (ppb) (0, 1, 5, 10, 20, 25 and 50 ppb) to establish a linear calibration curve for each element. Titanium-48 is the most prevalent form of titanium and was used for analysis. Iron-57 isotope was used to analyze the level of iron in the samples.

Data was collected with a SC-2 Autosampler (Elemental Scientific Inc., Omaha, NE, USA) and analyzed by the X-Series II ICP-MS (Thermo Scientific, Waltham, MA, USA) at the Northwestern University Quantitative Bioelemental Imaging Center.

### 4.8. Immunohistochemistry

To evaluate the proliferation rate of VX2 tumors, Ki67 antigen was quantitatively examined in VX2 slides. Conversely, Terminal deoxynucleotidyl transferase dUTP nick end labeling (TUNEL) assay was used to assess apoptotic cell death in tissue samples.

Consecutive tissue sections (4 μm thick) were cut and placed in a 58–60 °C oven overnight. For Ki67, deparaffinization, epitope retrieval, immunostaining and counter staining are done following Leica Bond Max and Polymer automated detection procedures. Slides were incubated in Bond DeWax solution at 72 °C and in epitope retrieval solution for 30 min at 100 °C. Slides were washed and incubated in primary antibody for 30 min, post primary polymer penetration enhancer reagent for 30 min, secondary antibody for 30 min and DAB reagent for 10 min is used with intermittent washes during the automated procedure. Antigen unmasking and endogenous peroxidase activity was quenched by incubation with 3% hydrogen peroxide for 10 min at room temperature by automated strainer. This work was done by the Pathology Core facility of Northwestern University.

The following antibodies were used for marker detection: For Ki67; Clone MIB-1 (Code M7240 Dako Denmark A/S Produktionsvej 42 DK-2600, Glostrup, Denmark); for TUNEL (S7101; Millipore, Billerica, MA, USA) was used. After DAB staining cell nuclei were counterstained with hematoxylin and blued in 0.4% ammonia water. Appropriate known control tissues were used as positive and negative control and primary Abs was omitted in “reagent delete” controls.

### 4.9. Semi-Quantitative Analysis of Ki67 and TUNEL Indices

Ki67 and TUNEL stained slides were scanned and the whole area of the tissue investigated. The expression of Ki67 (nuclear) was analyzed manually by light microscopy by pathology associate who was blinded for the treatment and control arms of the study. TUNEL staining was evaluated by counting positively stained pyknotic bodies (10,000 cells imaged per sample on average) in the viable VX2 tumor edge area, thereby excluding central necrotic areas of the tumors. Three random fields toward the invasive tumor edge were scored for TUNEL staining following the notion that the viable edge area of the tumor is the most biologically active and the most likely to drive the outcome of the disease [28].

## Figures and Tables

**Table 1:** Ratio of Ti vs. elements abundant in biological samples shows different accumulation of nanoparticles in different tissues when nanoparticle delivery by IV vs. IC is done. As a type of internal control ratio of Ti vs. Fe is shown as well. Due to the core–shell structure of nanoparticles these two elements have a constant ratio, and show no variation from sample to sample or tissue to tissue.

Elemental Content Ratio		Mean			SD	
		IC	IV	*p*	IC	IV
VX2	Ti/P *	0.01711	0.02127	0.26872	0.00297	0.00476
	Ti/S	0.01528	0.01383	0.84901	0.00224	0.01217
	Ti/Zn	0.64011	0.65397	0.89887	0.14092	0.10773
	Ti/Fe	0.12339	0.1504	0.27581	0.02431	0.02803
	Ti **	370.5783	394.69696	0.91055	261.26612	231.72104
	Fe	3362.35601	2735.3082	0.75481	2800.07347	1643.73117
Liver	Ti/P	0.01262	0.02182	0.03075 ***	0.00373	0.00313
	Ti/S	0.00736	0.0137	**0.00965**	0.0023	0.00052
	Ti/Zn	0.26631	0.45213	**0.02825**	0.08716	0.03959
	Ti/Fe	0.06452	0.09442	0.0991	0.02088	0.01223
	Ti	590.53869	953.44229	0.08483	48.8673	271.3826
	Fe	9580.52357	10,107.78307	0.7948	1985.14	2617.5
Spleen	Ti/P	0.0149	0.03427	**0.0403**	0.00564	0.00969
	Ti/S	0.01895	0.04221	**0.04122**	0.00697	0.01165
	Ti/Zn	0.85428	2.125	**0.03773**	0.50079	0.51691
	Ti/Fe	0.04296	0.08506	0.13063	0.0064	0.0379
	Ti	891.72723	1807.40153	0.09733	445.70119	445.70119
	Fe	20,612.158	22,297.20167	0.81604	10,397.89461	5469.26885
Lung	Ti/P	0.02079	0.02239	0.8848	0.01005	0.01489
	Ti/S	0.00803	0.01081	0.28208	0.00107	0.00373
	Ti/Zn	0.44638	0.61228	0.4479	0.1362	0.31355
	Ti/Fe	0.07881	0.08918	0.77821	0.04038	0.04388
	Ti	575.33695	490.11802	0.64474	143.85801	259.27169
	Fe	8816.3227	8795.4073	0.99758	4989.00812	10,081.30602
Kidney	Ti/P	0.01028	0.0146	0.11988	0.00219	0.0031
	Ti/S	0.00568	0.0087	0.10969	0.0018	0.0018
	Ti/Zn	0.20283	0.40091	**0.03195**	0.05167	0.09277
	Ti/Fe	0.07926	0.13716	0.06617	0.02248	0.03306
	Ti	374.46392	691.11448	0.04831	126.5038	148.62759
	Fe	4679.936	5062.48907	0.24386	266.53554	405.51323

* White, elemental ratios; ** gray, mean femtograms per pixel, based on Figure 5; *** bold, statistically significant difference between IC and IV NPs delivery, *p* < 0.05.

## Supplementary Materials

The following are available online at http://www.mdpi.com/2079-4991/6/8/143/s1, Figure S1: Cryo-TEM of nanoparticles deposited on lacey carbon TEM grid, Figure S2: (a) Two VX2 tumors implanted in left lobe of rabbit liver after gross necropsy; (b) Representative selective hepatic artery X-ray DSA contrast in rabbit liver showing hepatic artery distribution (b-1) and VX2 tumors perfusion (b-2), Figure S3: Side by side comparison of tissue overview images obtained with H&E staining and histochemical staining for nanoparticles based on dopamine attachment to nanoparticles in situ. Details of these images are presented in Figure 2 and Figure 3.

## Abbreviations

The following abbreviations are used in this manuscript:

ICP-MS	Inductively coupled plasma mass spectrometry
XFM	X-ray fluorescence microscopy
