# Supplementary material for: Distribution of Iron Oxide Core-Titanium Dioxide Shell Nanoparticles in VX2 Tumor Bearing Rabbits Introduced by Two Different Delivery Modalities
Source: Nanomaterials (Basel). 2016 Aug 3;6(8):143. doi: 10.3390/nano6080143 (PMC5224625; doi:10.3390/nano6080143)
Supplement: Supplementary file 1 [file nanomaterials-06-00143-s001.pdf]

Supplementary Materials: Distribution of Iron Oxide Core-Titanium Dioxide Shell Nanoparticles in VX2 Tumor Bearing Rabbits Introduced by Two Different Delivery Modalities

Tamer Refaat, Derek West, Samar El Achy, Vamsi Parimi, Jasmine May, Lun Xin, Kathleen R. Harris, William Liu, Michael Beau Wanzer, Lydia Finney, Evan Maxey, Stefan Vogt, Reed A. Omary, Daniele Procissi, Andrew C. Larson, Tatjana Paunesku and Gayle E. Woloschak

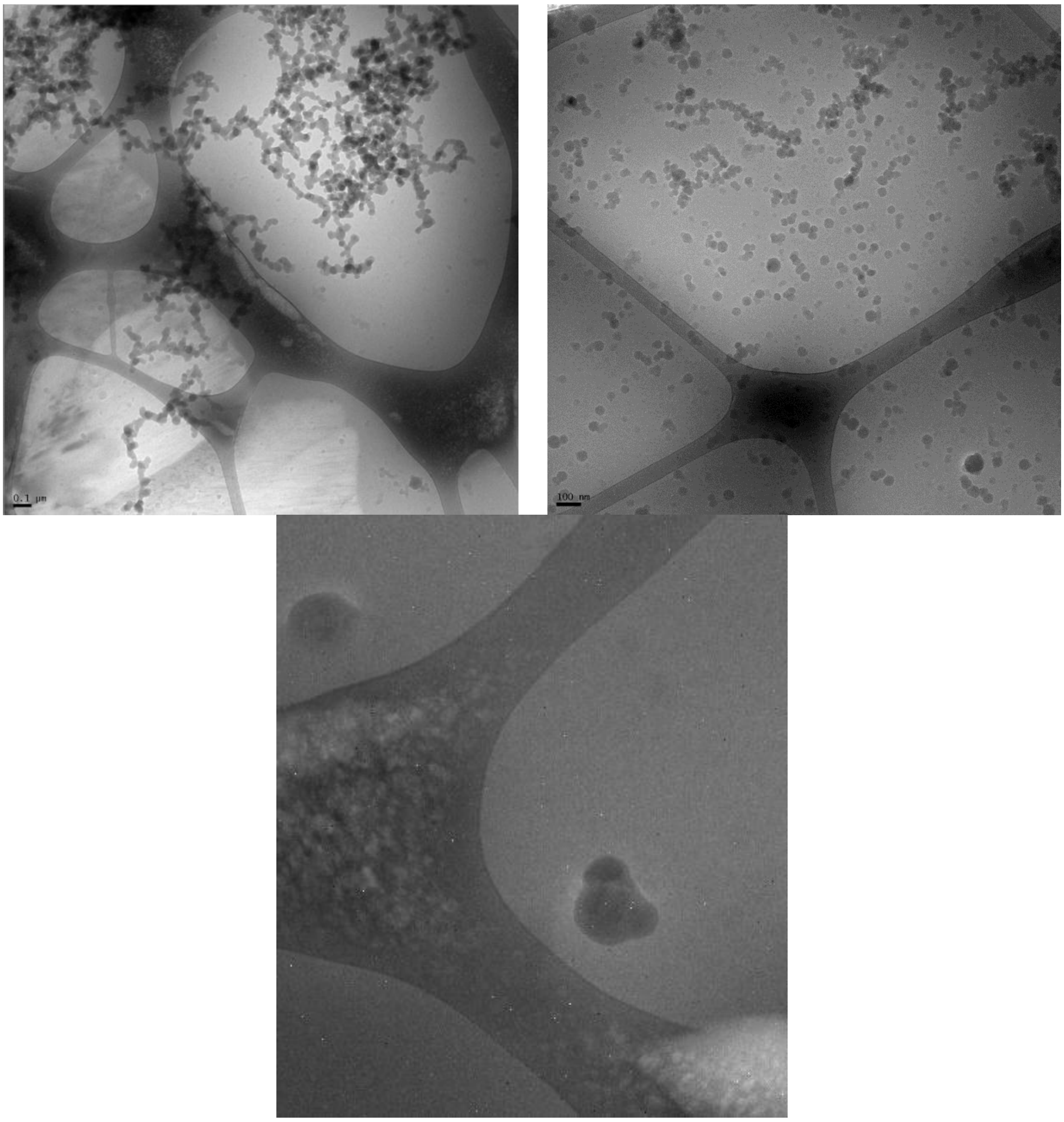

Figure S1. Cryogenic transmission electron microscopy (Cryo-TEM) of nanoparticles deposited onto and frozen on lacey carbon TEM grid at magnification: (**Top left**) 6000×; and (**top right**) 8000×. (**Bottom Left**) Excerpt from a 10,000× image of the same grid. Slightly darker areas of the nanoparticle correspond to iron oxide core particles. Overall, shapes and sizes of both core nanoparticles and core-shell nanoparticles were variable. This is in keeping with results of colloidal TiO_2 synthesis we have done in the past, both for pure TiO_2 nanoparticles and for particles containing Fe_3O_4 shells [1–13].

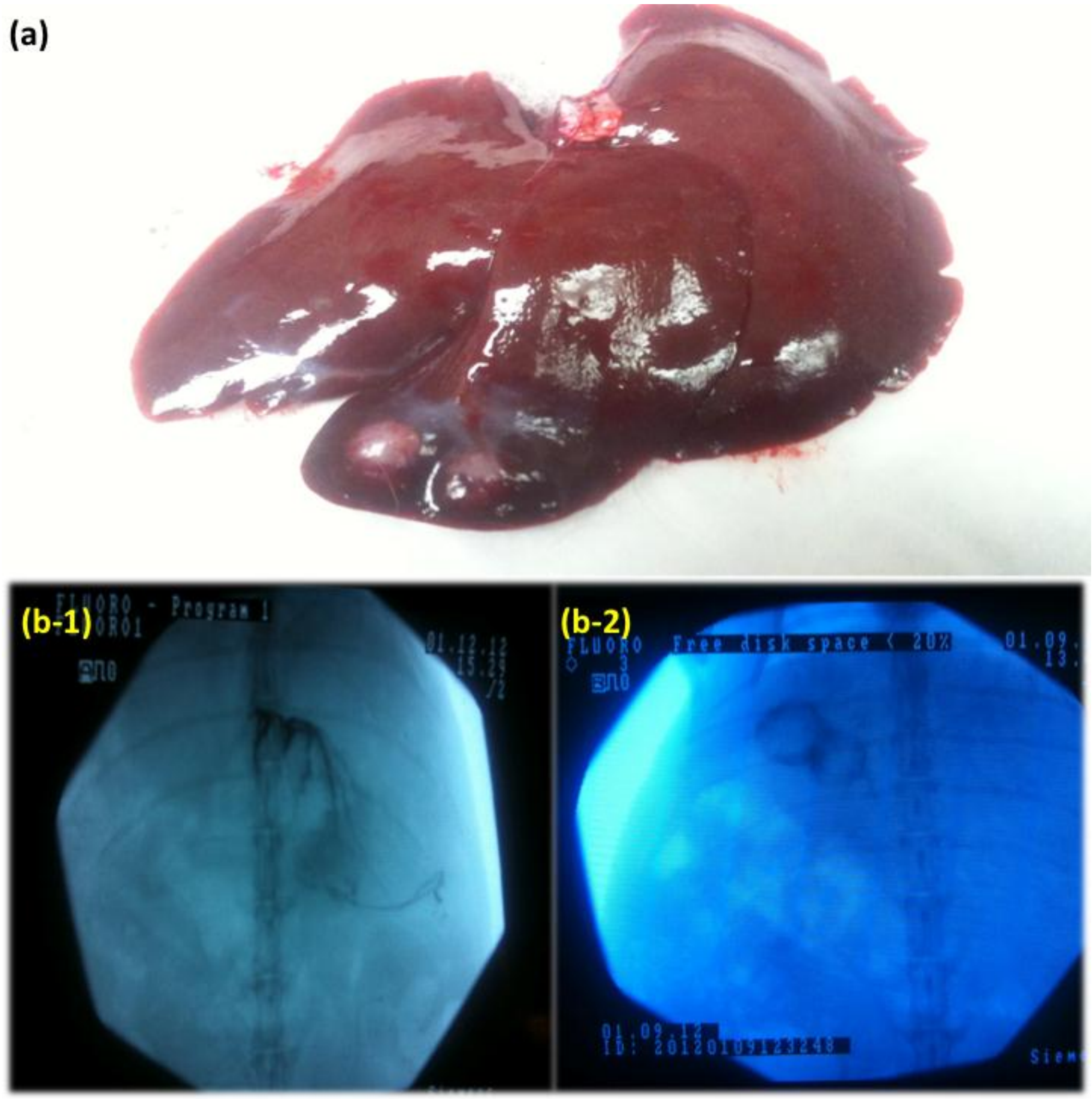

Figure S2. (**a**) Two VX2 tumors implanted in left lobe of rabbit liver after gross necropsy. (**b**) Representative selective hepatic artery X-ray DSA contrast in rabbit liver showing: hepatic artery distribution (**b-1**); and VX2 tumors perfusion (**b-2**). We have used X-Ray digital subtraction angiography (DSA) imaging to help us guide transarterial intra-catheter delivery to liver tumors as has been done in the past.

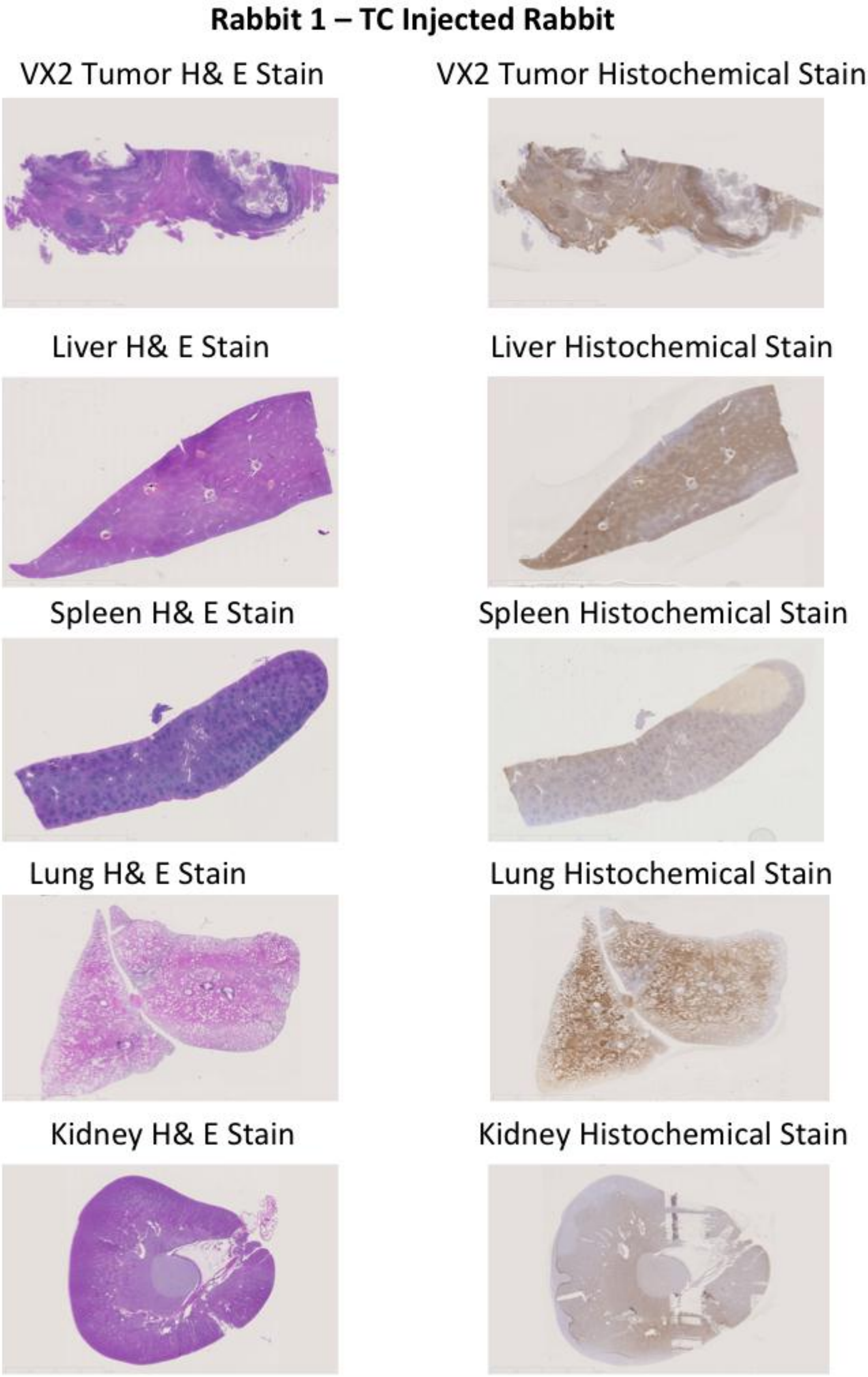
Rabbit 1 – TC Injected Rabbit
VX2 Tumor H& E Stain
VX2 Tumor Histochemical Stain
Liver H& E Stain
Liver Histochemical Stain
Spleen H& E Stain
Spleen Histochemical Stain
Lung H& E Stain
Lung Histochemical Stain
Kidney H& E Stain
Kidney Histochemical Stain

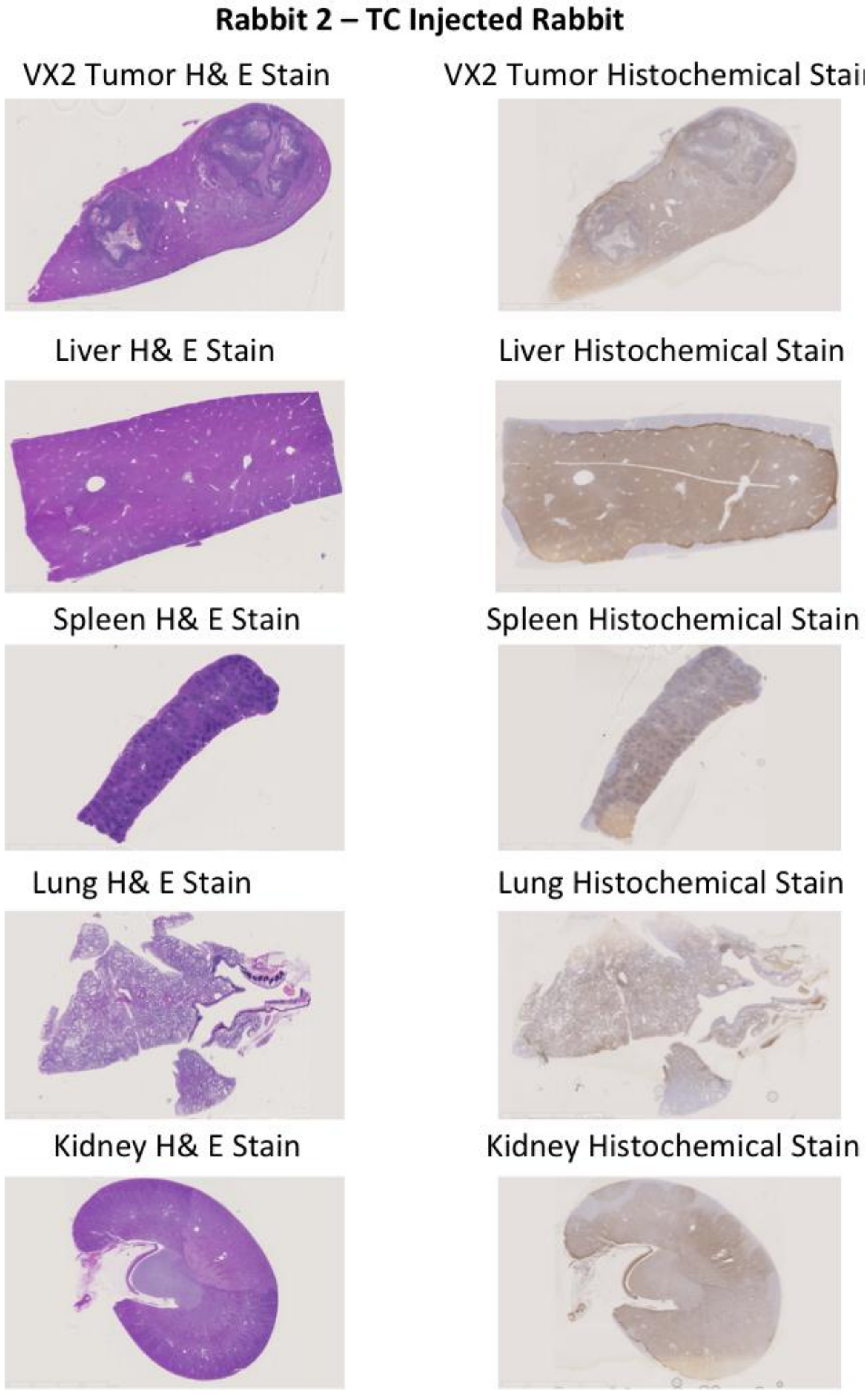
Rabbit 2 – TC Injected Rabbit
VX2 Tumor H& E Stain
VX2 Tumor Histochemical Stai
Liver H& E Stain
Liver Histochemical Stain
Spleen H& E Stain
Spleen Histochemical Stain
Lung H& E Stain
Lung Histochemical Stain
Kidney H& E Stain
Kidney Histochemical Stain

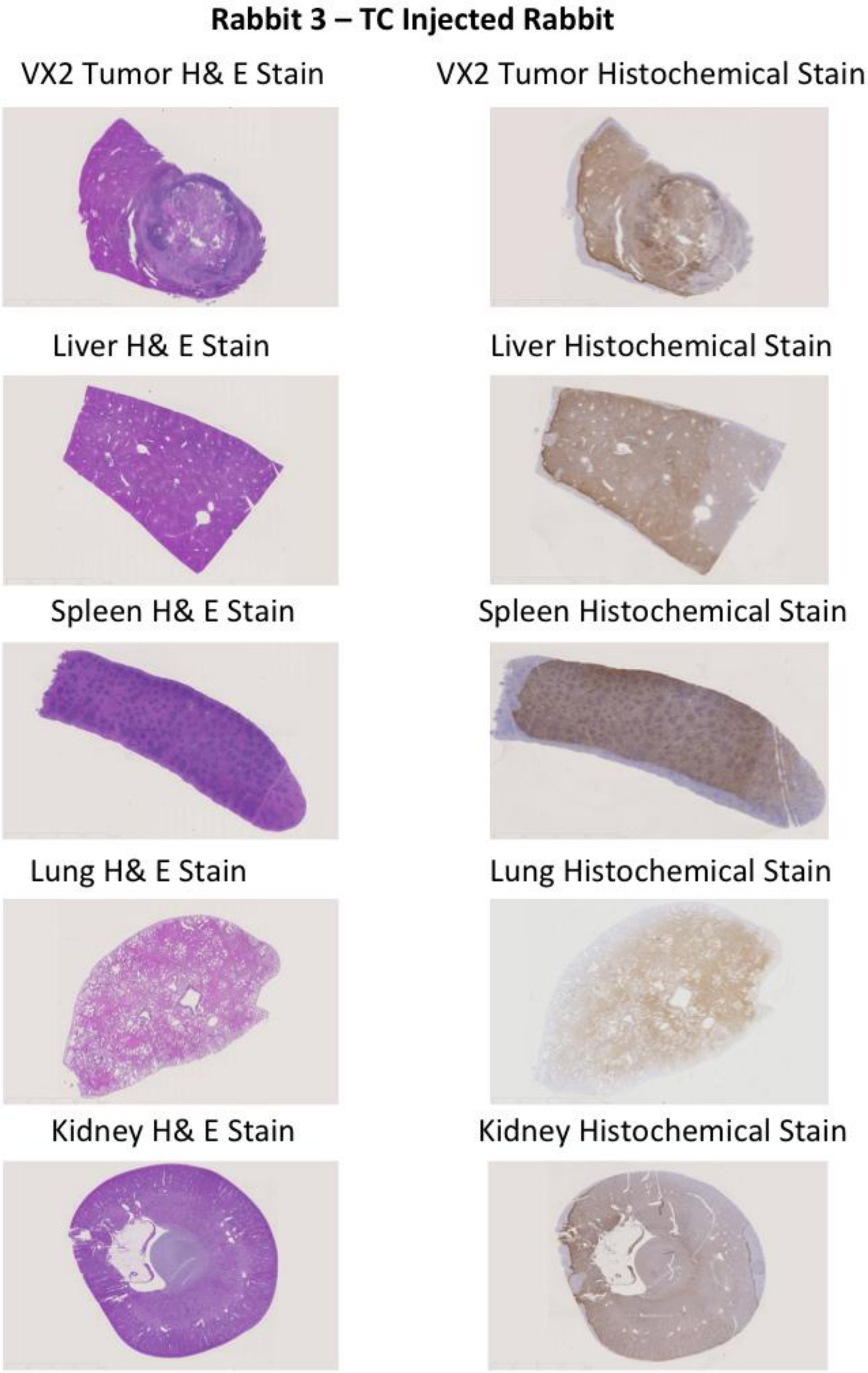
Rabbit 3 – TC Injected Rabbit
VX2 Tumor H& E Stain
VX2 Tumor Histochemical Stain
Liver H& E Stain
Liver Histochemical Stain
Spleen H& E Stain
Spleen Histochemical Stain
Lung H& E Stain
Lung Histochemical Stain
Kidney H& E Stain
Kidney Histochemical Stain

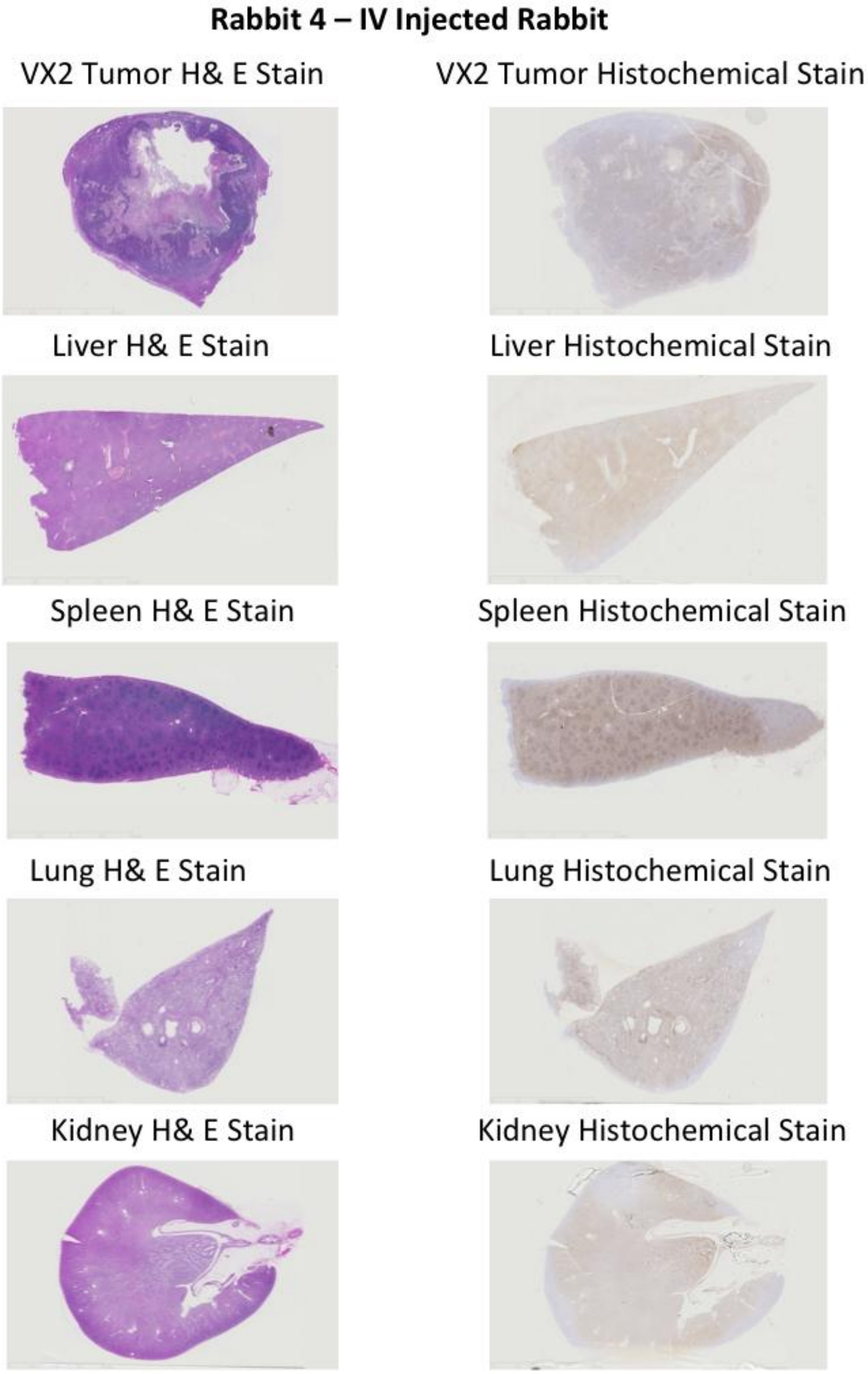
Rabbit 4 – IV Injected Rabbit
VX2 Tumor H& E Stain
VX2 Tumor Histochemical Stain
Liver H& E Stain
Liver Histochemical Stain
Spleen H& E Stain
Spleen Histochemical Stain
Lung H& E Stain
Lung Histochemical Stain
Kidney H& E Stain
Kidney Histochemical Stain

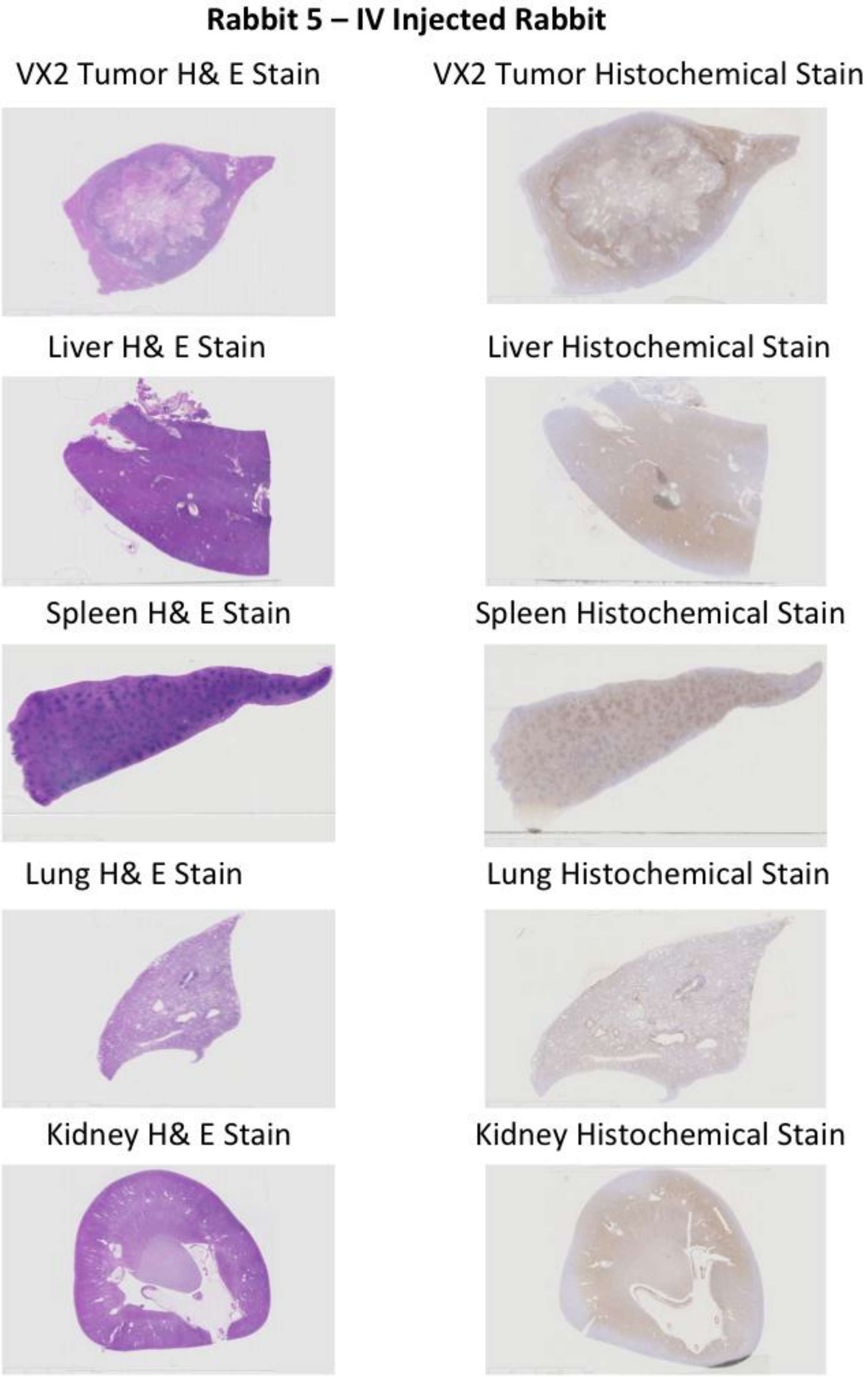
Rabbit 5 – IV Injected Rabbit
VX2 Tumor H& E Stain
VX2 Tumor Histochemical Stain
Liver H& E Stain
Liver Histochemical Stain
Spleen H& E Stain
Spleen Histochemical Stain
Lung H& E Stain
Lung Histochemical Stain
Kidney H& E Stain
Kidney Histochemical Stain

Rabbit 6 – IV Injected Rabbit

VX2 Tumor H& E Stain

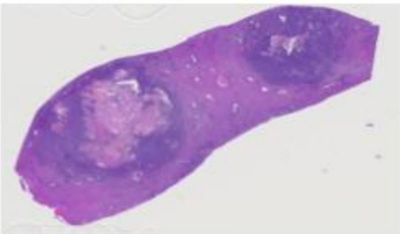

VX2 Tumor Histochemical Stain

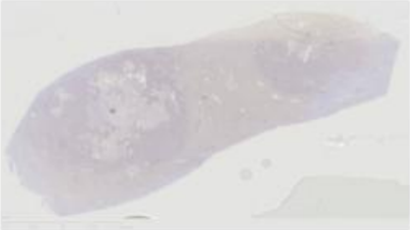

Liver H& E Stain

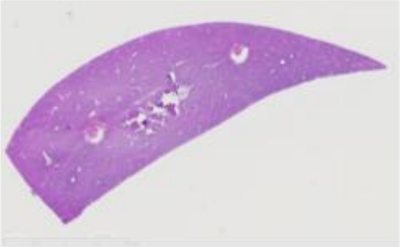

Liver Histochemical Stain

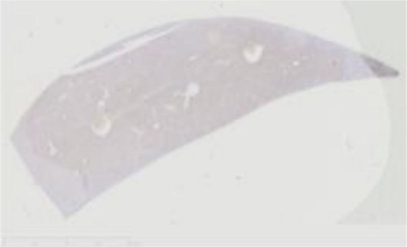

Spleen H& E Stain

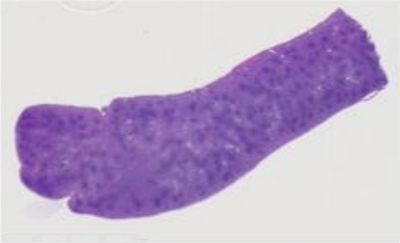

Spleen Histochemical Stain

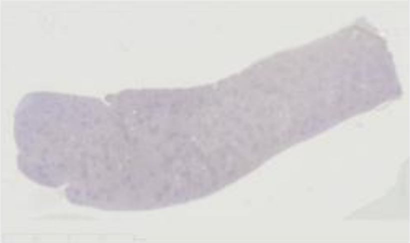

Lung H& E Stain

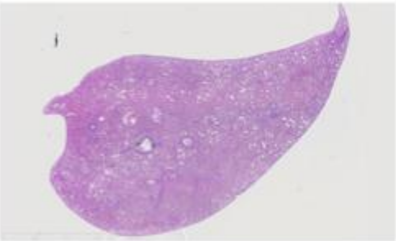

Lung Histochemical Stain

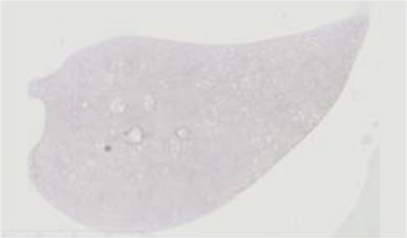

Kidney H& E Stain

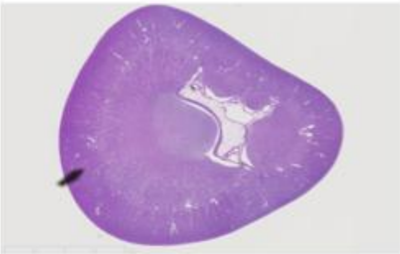

Kidney Histochemical Stain

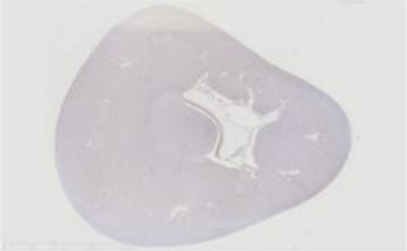

Control 1 - Not Injected Rabbit

Liver H& E Stain

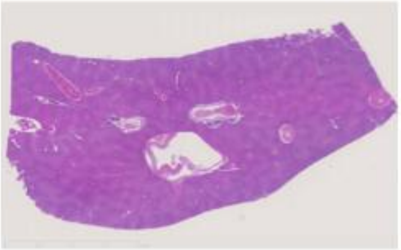

Liver Histochemical Stain

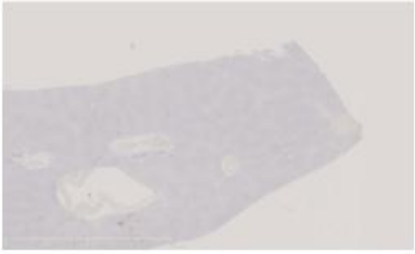

Spleen H& E Stain

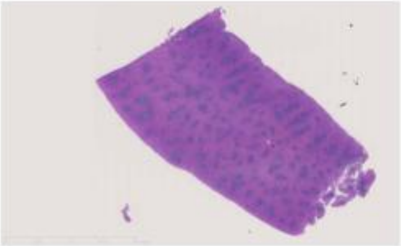

Spleen Histochemical Stain

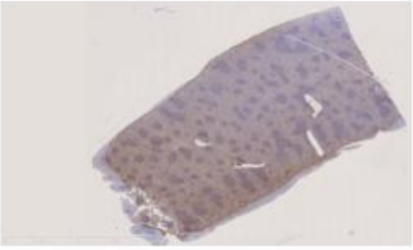

Lung H& E Stain

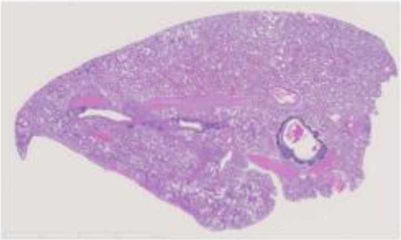

Lung Histochemical Stain

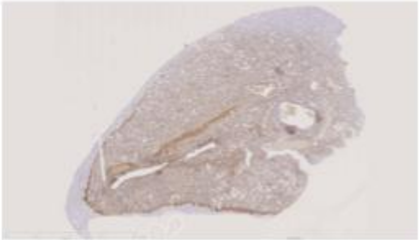

Kidney H& E Stain

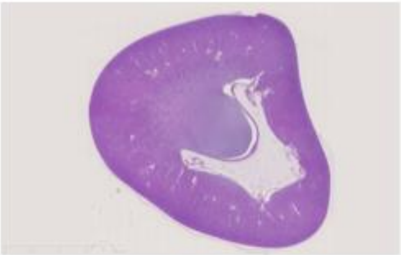

Kidney Histochemical Stain

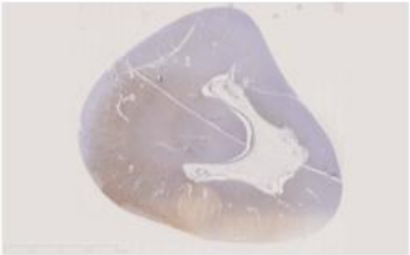

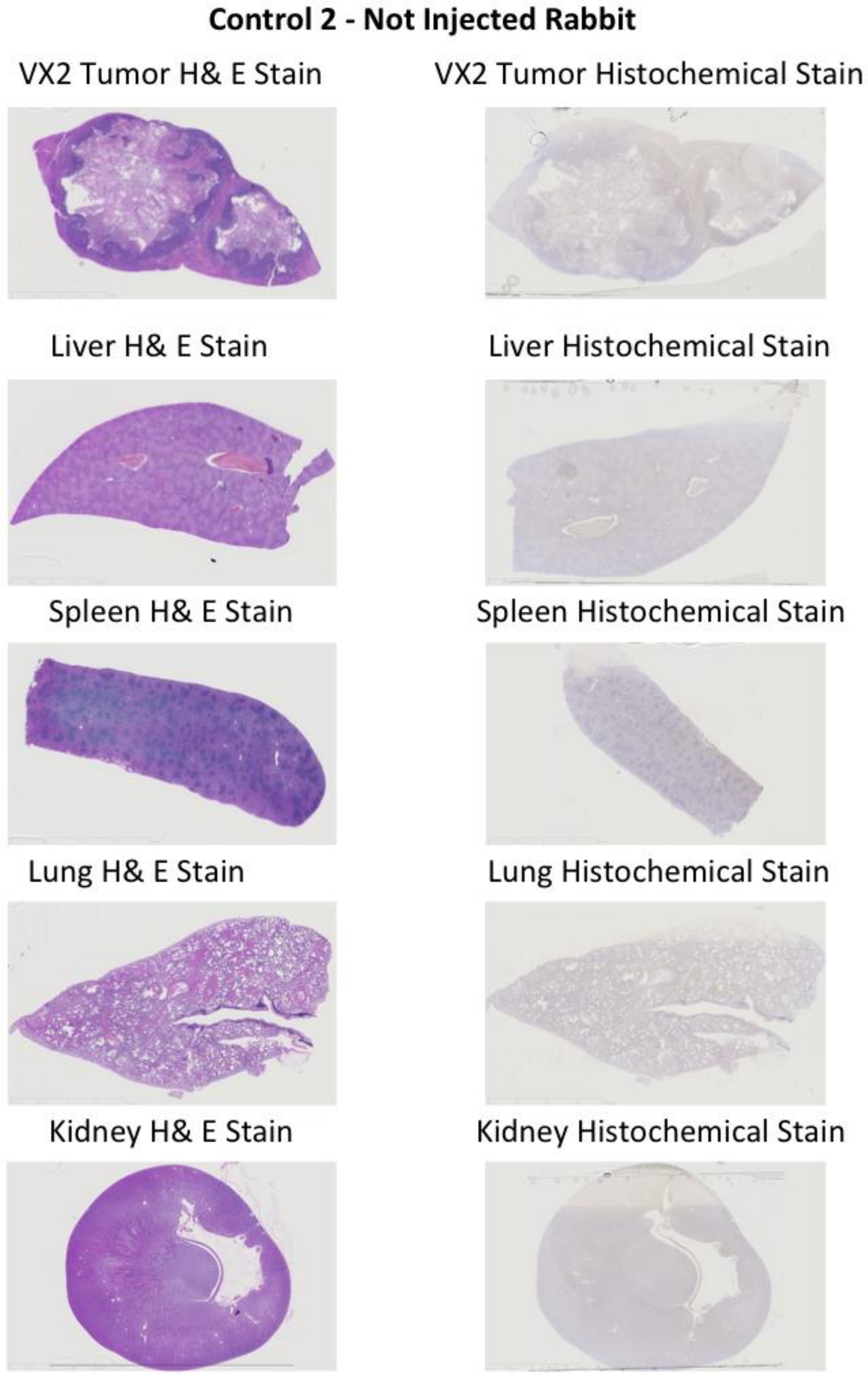

Figures S3. Side by side comparison of tissue overview images obtained with hematoxylin and eosin (H&E) staining and histochemical staining for nanoparticles based on dopamine attachment to nanoparticles in situ. Details of these images are presented in Figures 2 and 3.

Reference

1. Arora, H.C.; Jensen, M.P.; Yuan, Y.; Wu, A.; Vogt, S.; Paunesku, T.; Woloschak, G.E. Nanocarriers enhance Doxorubicin uptake in drug-resistant ovarian cancer cells. *Cancer Res*. **2012**, *72*, 769–778.
2. Bazak, R.; Ressl, J.; Raha, S.; Doty, C.; Liu, W.; Wanzer, B.; Salam, S.A.; Elwany, S.; Paunesku, T.; Woloschak, G.E. Cytotoxicity and DNA cleavage with core-shell nanocomposites functionalized by a KH domain DNA binding peptide. *Nanoscale* **2013**, *5*, 11394–11399.
3. Brown, E.M.; Paunesku, T.; Wu, A.; Thurn, K.T.; Haley, B.; Clark, J.; Priester, T.; Woloschak, G.E. Methods for assessing DNA hybridization of peptide nucleic acid-titanium dioxide nanoconjugates. *Anal. Biochem*. **2008**, *383*, 226–235.
4. Endres, P.J.; Paunesku, T.; Vogt, S.; Meade, T.J.; Woloschak, G.E. DNA-TiO_2 nanoconjugates labeled with magnetic resonance contrast agents. *J. Am. Chem. Soc.* **2007**, *129*, 15760–15761.
5. Kurepa, J.; Paunesku, T.; Vogt, S.; Arora, H.; Rabatic, B.M.; Lu, J.; Wanzer, M.B.; Woloschak, G.E.; Smalle, J.A. Uptake and distribution of ultrasmall anatase TiO_2 Alizarin red S nanoconjugates in Arabidopsis thaliana. *Nano Lett*. **2010**, *10*, 2296–2302.
6. Paunesku, T.; Ke, T.; Dharmakumar, R.; Mascheri, N.; Wu, A.; Lai, B.; Vogt, S.; Maser, J.; Thurn, K.; Szolc-Kowalska, B. Gadolinium-conjugated TiO_2-DNA oligonucleotide nanoconjugates show prolonged intracellular retention period and T1-weighted contrast enhancement in magnetic resonance images. *Nanomed. Nanotechnol. Biol. Med*. **2008**, *4*, 201–207.
7. Paunesku, T.; Rajh, T.; Wiederrecht, G.; Maser, J.; Vogt, S.; Stojicevic, N.; Protic, M.; Lai, B.; Oryhon, J.; Thurnauer, M. Biology of TiO_2-oligonucleotide nanocomposites. *Nat. Mater*. **2003**, *2*, 343–346.
8. Paunesku, T.; Vogt, S.; Lai, B.; Maser, J.; Stojicevic, N.; Thurn, K.T.; Osipo, C.; Liu, H.; Legnini, D.; Wang, Z. Intracellular distribution of TiO_2-DNA oligonucleotide nanoconjugates directed to nucleolus and mitochondria indicates sequence specificity. *Nano Lett*. **2007**, *7*, 596–601.
9. Paunesku, T.; Wanzer, M.B.; Kirillova, E.N.; Muksinova, K.N.; Revina, V.S.; Lyubchansky, E.R.; Grosche, B.; Birschwilks, M.; Vogt, S. X-ray fluorescence microscopy for investigation of archival tissues. *Health Phys*. **2012**, *103*, 181–186.
10. Thurn, K.T.; Arora, H.; Paunesku, T.; Wu, A.; Brown, E.M.; Doty, C.; Kremer, J.; Woloschak, G. Endocytosis of titanium dioxide nanoparticles in prostate cancer PC-3M cells. *Nanomed. Nanotechnol. Boil. Med*. **2011**, *7*, 123–130.
11. Thurn, K.T.; Paunesku, T.; Wu, A.; Brown, E.M.; Lai, B.; Vogt, S.; Maser, J.; Aslam, M.; Dravid, V.; Bergan, R. Labeling TiO_2 nanoparticles with dyes for optical fluorescence microscopy and determination of TiO_2-DNA nanoconjugate stability. *Small* **2009**, *5*, 1318–1325.
12. Wu, A.; Paunesku, T.; Brown, E.M.; Babbo, A.; Cruz, C.; Aslam, M.; Dravid, V.; Woloschak, G.E. Titantium Dioxide Nanoparticles Assembled by DNA Molecules Hybridization and Loading of DNA Interacting Proteins. *Nano* **2008**, *3*, 27–36.
13. Yuan, Y.; Chen, S.; Paunesku, T.; Gleber, S.C.; Liu, W.C.; Doty, C.B.; Mak, R.; Deng, J.; Jin, Q.; Lai, B. Epidermal growth factor receptor targeted nuclear delivery and high-resolution whole cell X-ray imaging of Fe_3O_4@TiO_2 nanoparticles in cancer cells. *ACS Nano* **2013**, *7*, 10502–10517.

© 2016 by the authors. Submitted for possible open access publication under the terms and conditions of the Creative Commons Attribution (CC-BY) license (http://creativecommons.org/licenses/by/4.0/).
